# Supplementary material for: A Comparative Analysis of Deep Learning Models for Automated Cross-Preparation Diagnosis of Multi-Cell Liquid Pap Smear Images
Source: Diagnostics (Basel). 2022 Jul 29;12(8):1838. doi: 10.3390/diagnostics12081838 (PMC9406372; doi:10.3390/diagnostics12081838)
Supplement: Supplementary file 1 [file diagnostics-12-01838-s001.zip › diagnostics-1759938-supplementary.pdf]

A Comparative Analysis of Deep Learning Models For Automated Cross-Preparation Diagnosis of Multi-Cell Liquid Pap Smear Images

Yasmin Karasu Benyes[1], E. Celeste Welch[1], Abhinav Singhal[2], Joyce Ou[3], Anubhav Tripathi[1]
[1]Center for Biomedical Engineering, School of Engineering, Brown University, Providence, RI, USA.
[2]Department of Computer Science and Engineering, Indian Institutes of Technology Delhi, Hauz Khas, New Delhi, India.
[3]Department of Pathology and Laboratory Medicine, Alpert Medical School, Brown University, Providence, RI, USA.

Supporting Documents

Supplemental Figure S1: Accuracy and loss plots for training data for basic CNN model.

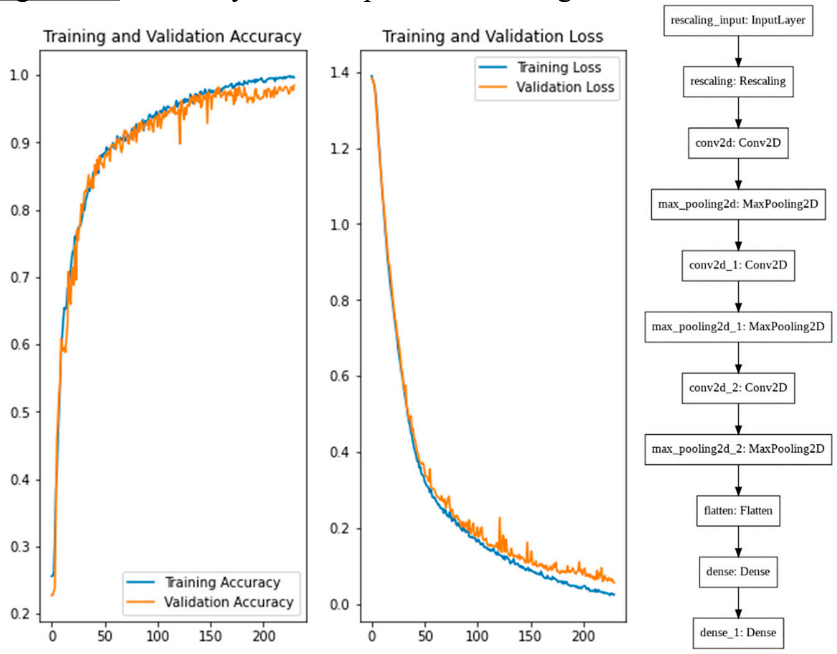

Supplemental Figure S2: Accuracy and loss plots for training data for AE CNN model.

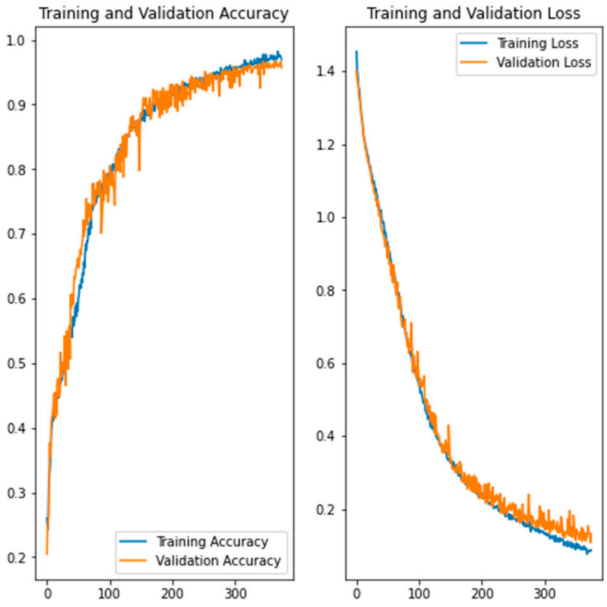

Supplemental Figure S3: Accuracy and loss plots for training data for best-performing single transfer model, ResNet50.

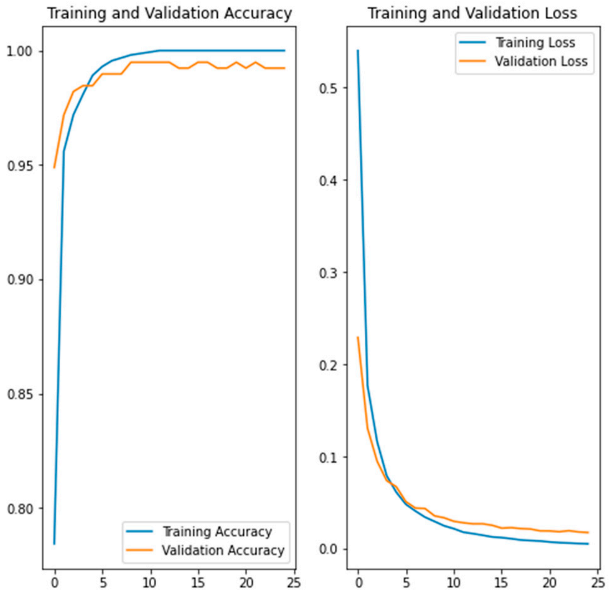

Supplemental Figure S4: Confusion matrix and classification reports for DenseNet121.

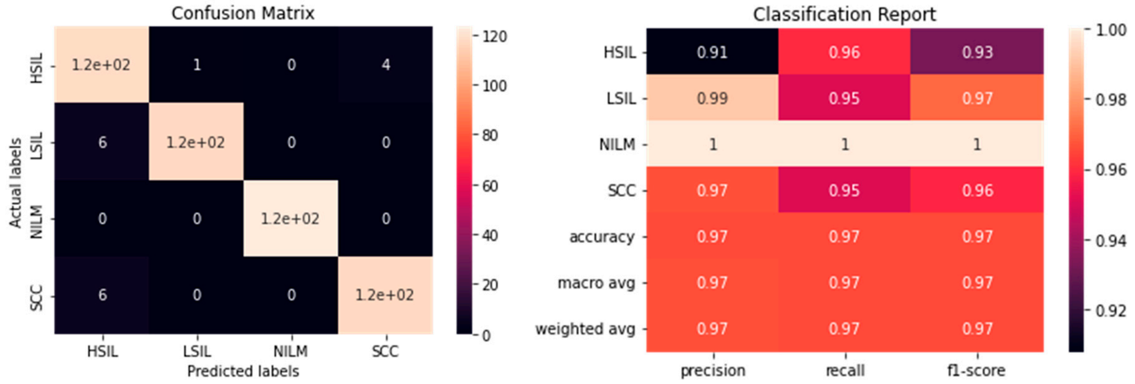

Supplemental Figure S5: Confusion matrix and classification report for ResNet101.

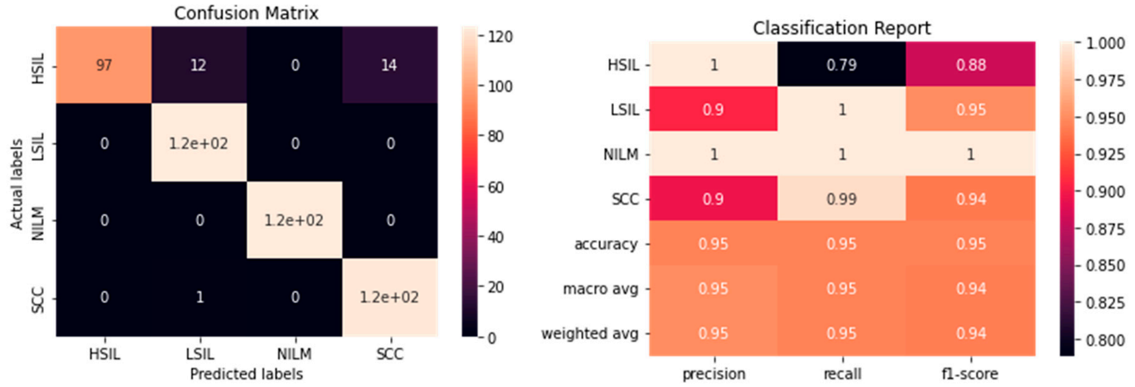

Supplemental Figure S6: Confusion matrix and classification report for ResNet152.

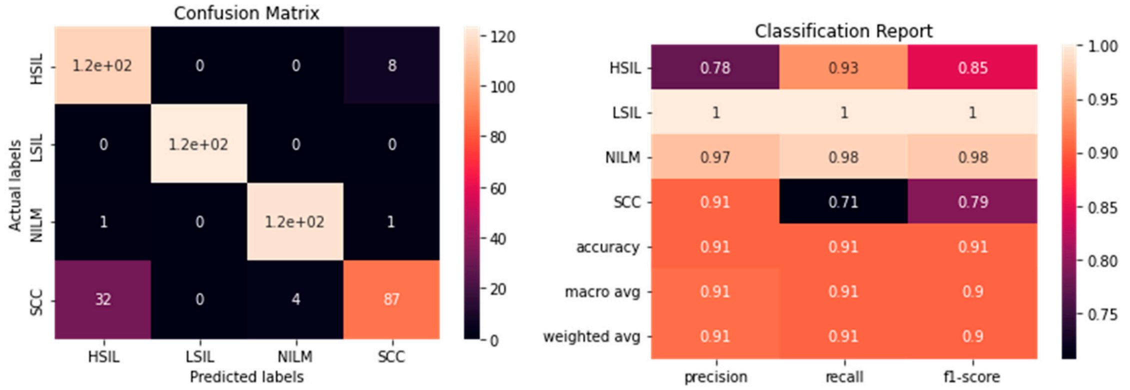

Supplemental Figure S7: Confusion matrix and classification report for EfficientNetB0.

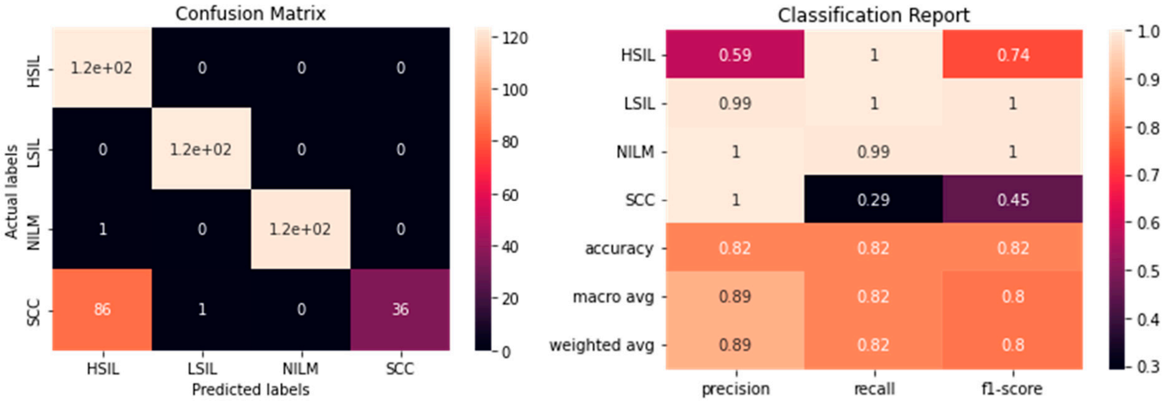

Supplemental Table S1: Chi-square, P-value, T-value and standard deviations for each tested model.

Model	Chi-Square	P-value	T-value	SD
CNN	58.17	<0.0001	51.62	17.32
CNN+ AE	29.71	<0.0001	100.8	9.256
ResNet50	4.98	0.1733	532.4	1.826
DenseNet121	17.23	0.0006	132.4	7.047
ResNet101	24.64	<0.0001	84.7	10.66
ResNet152	67.41	<0.0001	36.34	23.28
EfficientNetB0	21.21	<0.0001	41.41	17.75
Ensemble	10.5	0.0148	274.2	3.512

Supplemental Figure S8: Plot of T-values for each tested model.

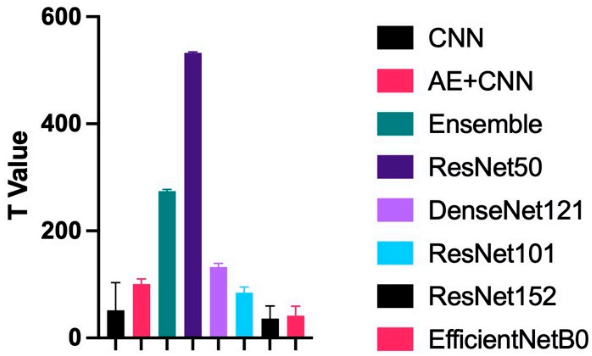

Supplemental Figure S9: Evaluation of the training and testing runtime for each model included in the analysis.

**Model**	**Training Runtime (For 1959 Samples), Minutes**	**Testing Runtime Per Sample, Minutes**
CNN Model	18.55899691581726	0.09384679794311523
AE CNN	580.802036523819 + 130.64781999588013	0.17501425743103027
ResNet50	105.51053142547607	0.8113045692443848
DenseNet121	114.49975991249084	2.1196017265319824
ResNet101	180.21313786506653	1.648026466369629
ResNet152	245.04961323738098	2.454149007797241
EfficientNetB0	86.03872275352478	1.2509117126464844
Ensemble	-	7.7521045207977295

Supplemental Figure S10: Information on epoch number, learning rate, activation function, dropout, and bath size for each model included in the analysis.

**Model**	**Epoch**	**Learning Rate**	**Activation Function**	**Dropout**	**Batch Size**
CNN Model	230	5e-5	relu	-	16
AE CNN	100 + 375	5e-5	relu/sigmoid	0.2	16
ResNet50	25	0.01	relu	-	32
DenseNet121	25	0.01	softmax	-	32
ResNet101	25	0.01	relu	-	32
ResNet152	25	0.01	sigmoid	-	32
EfficientNetB0	25	0.01	softmax	-	32
Ensemble	-	-	-	-	-
